# Dramatic Response to Pyrotinib and T-DM1 in HER2-Negative Metastatic Breast Cancer With 2 Activating *HER2* Mutations

**DOI:** 10.1093/oncolo/oyad122

**Published:** 2023-04-29

**Authors:** Hao Tian, Man Qu, Guozhi Zhang, Long Yuan, Qiyun Shi, Yinhuan Wang, Ying Yang, Yi Zhang, Xiaowei Qi

**Affiliations:** Department of Breast and Thyroid Surgery, Southwest Hospital, Army Medical University, Chongqing, People’s Republic of China; Department of Breast and Thyroid Surgery, Southwest Hospital, Army Medical University, Chongqing, People’s Republic of China; Department of Breast and Thyroid Surgery, Southwest Hospital, Army Medical University, Chongqing, People’s Republic of China; Department of Breast and Thyroid Surgery, Southwest Hospital, Army Medical University, Chongqing, People’s Republic of China; Department of Breast and Thyroid Surgery, Southwest Hospital, Army Medical University, Chongqing, People’s Republic of China; Department of Breast and Thyroid Surgery, Southwest Hospital, Army Medical University, Chongqing, People’s Republic of China; Department of Breast and Thyroid Surgery, Southwest Hospital, Army Medical University, Chongqing, People’s Republic of China; Department of Breast and Thyroid Surgery, Southwest Hospital, Army Medical University, Chongqing, People’s Republic of China; Department of Breast and Thyroid Surgery, Southwest Hospital, Army Medical University, Chongqing, People’s Republic of China

**Keywords:** breast cancer, HER2 mutation, pyrotinib, T-DM1

## Abstract

HER2 signaling is activated in response to somatic *HER2* mutations, which are often found in invasive lobular breast cancer (ILC) and are associated with poor prognosis. Tyrosine kinase inhibitors (TKIs) have demonstrated considerable antitumor activity in patients with *HER2*-mutated advanced breast cancer (BC). Further, several clinical trials have indicated that HER2-targeted antibody-drug conjugates (ADCs) exhibit promising efficacy in lung cancer with *HER2* mutations, and the efficacy of ADCs against *HER2*-mutated BC is currently being evaluated. Several preclinical studies have demonstrated that the therapeutic efficacy of ADCs in *HER2*-mutated cancer can be enhanced by the addition of irreversible TKIs, but the potential of such a combined treatment regimen for the treatment of *HER2*-mutated BC has not been reported. Herein, we describe a case in which a patient with estrogen receptor-positive/HER2-negative metastatic ILC with 2 activating *HER2* mutations (D769H and V777L) exhibited a significant and durable response to anti-HER2 treatment with pyrotinib (an irreversible TKI) in combination with ado-trastuzumab emtansine, which was administered after multiple lines of therapy that had resulted in disease progression. Further, based on the evidence from the present case, TKI plus ADC seems to be a promising combination anti-HER2 regimen for patients with HER2-negative/*HER2*-mutated advanced BC, although further rigorous studies are warranted to confirm these findings.

## Teaser

This case report is the first to describe the treatment of invasive lobular breast cancer carrying HER2 mutations with combined treatment involving the tyrosine kinase inhibitor pyrotinib and the antibody-drug conjugate ado-trastuzumab emtansine. The regimen was promising.

**Key Points**

- Patients with breast cancer (BC) with *HER2*-activating mutations may benefit from anti-HER2 therapy, especially irreversible TKI and ADC (ongoing trial), while trials have confirmed that the clinical activity of single-target therapy (TKI) in treating *HER2*-mutated breast cancer is limited by various factors.
- Preclinical studies showed that irreversible TKI can enhance the antitumor activity of ADC, which indicate that combined treatment with TKIs and ADCs in BC with *HER2* mutations is worthy of further exploration.
- The present report is the first to describe the treatment of MBC carrying *HER2* activating mutations with combined treatment involving TKI and ADC.

## Introduction

In patients with advanced breast cancer (BC), the posterior-line treatment stage is particularly challenging. However, with the increase in studies on genomic profiling and the clinical application of NGS, the management of advanced BC has already entered the era of precision treatment. As a result, it is possible to explore and design personalized treatment strategies according to the gene alteration patterns of individual patients.

The *HER2* gene was first discovered and cloned in 1983.^1^ Amplification of the *HER2* gene results in activation of the HER2 signaling pathway, which is closely related to the occurrence and poor prognosis of breast cancer.^2,3^*HER2* mutations were preliminarily described as oncogenic drivers based on single-strand conformational polymorphism assay and NGS in 2006 and 2009, respectively.^4,5^*HER2* somatic mutations can drive tumor progression by activating the HER2 signaling pathway, but the frequency of somatic *HER2* mutations is lower than that of *HER2* amplification, and the incidence of *HER2* somatic mutations is 2%-4% in breast cancer.^4,6,7^ Somatic *HER2* mutations are more likely to occur in patients with HER2-negative BC or BC with low HER2 expression, and these mutations are associated with a lower survival rate in patients.^8-10^

Ross et al found that the co-occurrence of *HER2* amplification and *HER2* mutation in recurrent and metastatic BC was 0.7%.^11^ Interestingly, HER2 positivity and *HER2* mutation are probably mutually exclusive in primary tumors and co-occur more often in metastatic lesions.^12,13^*HER2* mutations have been shown to be associated with invasive lobular breast cancer (ILC), which has a *HER2* mutation rate of 5.1%.^14^ Furthermore, in patients with *CDH1*-mutated ILC, somatic *HER2* mutation co-occurs more frequently, particularly in metastases (15% in ILC versus 5% in invasive BC of no specific type).^8,15,16^ HER2-targeted compounds for HER2-positive BC consist of monoclonal antibodies (trastuzumab and pertuzumab), tyrosine kinase inhibitors (TKIs), and antibody-drug conjugates (ADCs), of which trastuzumab is usually considered a cornerstone and first-line drug. However, trastuzumab usually fails to effectively suppress HER2-negative/*HER2*-mutant BC, since the expression level of HER2 in HER2-negative BC is relatively low and 70%-90% of *HER2* mutations occur in the kinase domain.^7,8,11^TKIs are well-established anti-HER2 agents for the treatment of metastatic BC with HER2 overexpression or *HER2* gene amplification.^17^ Similarly, TKIs, especially one of the most studied irreversible TKI neratinib, also demonstrated considerable activity against advanced BC with *HER2* mutations in in vitro and clinical studies, but the response rate and clinical benefit rate were still lower than those of anti-HER2 therapy in *HER2*-amplified BC.^8,18-20^ An ongoing phase II clinical trial has been designed to explore the efficacy of TKI tucatinib plus trastuzumab ±fulvestrant in *HER2*-mutated metastatic BC and other malignant solid tumors (NCT04579380).

The use of ADCs, including T-DM1 and trastuzumab deruxtecan (T-DXd), has been described in the treatment of *HER2-*mutated cancer, especially lung cancer. In a study involving 18 patients with *HER2*-mutated advanced lung adenocarcinomas, a response rate of 44% and a median progression-free survival (PFS) of 5 months was observed for T-DM1.^21^ Furthermore, the DESTINY-Lung 01 trial enrolled 91 metastatic *HER2*-mutated non-small cell lung cancer (NSCLC) patients whose tumors were refractory to standard therapy, and the results demonstrated that T-DXd showed durable anticancer activity with a median PFS of 9.3 months.^22^ Up to now, no clinical trial has been designed to specifically examine the efficacy of ADCs in treating *HER2*-mutant BC. However, Li and team, who initiated the DESTINY-Lung 01 trial, have included *HER2*-mutated/HER2-negative metastatic BC in their ongoing clinical trial on the efficacy and safety of T-DXd for the treatment of advanced solid tumors with activating *HER2* mutations (DESTINY-PanTumor01 trial number NCT04639219).

Pyrotinib, an irreversible pan-HER tyrosine kinase inhibitor against HER1, HER2, and HER4, has demonstrated promising anticancer activity in HER2-positive metastatic BC, and the results of a multicenter phase III trial (PHOBE) indicated that pyrotinib plus capecitabine significantly improved PFS in HER2-positive metastatic BC, as compared to lapatinib plus capecitabine.^23^ In *HER2*-mutated cancer settings, pyrotinib has shown stronger antitumor activity than afatinib and T-DM1 in *HER2*-mutated NSCLC patient-derived organoid or xenograft models, and pyrotinib monotherapy exhibited promising activity in 60 unresectable stages III or IV NSCLC patients with an objective response rate of 30% and a median overall survival of 14.4 months.^24,25^

Most recently, Kaplan et al reported a patient with metastatic ER-positive, *HER2*-nonamplified ILC with an activating *HER2* mutation (*HER2* p780_y781insGSP) whose tumor became resistant to neratinib as well as capecitabine, but whose subsequent leptomeningeal disease exhibited a dramatic response to tucatinib plus capecitabine with a PFS of 10 months.^26^ The past, future, and immense potential of treating *HER2*-mutated BC with TKI alone or in combination with ADCs have also been thoroughly reviewed and discussed in their case report. Based on the available evidence, it appears that the treatment of *HER2*-mutant advanced BC with various TKIs has been previously evaluated with promising outcomes. In addition, ADCs may have potential for the treatment of *HER2*-mutated BC based on their robust activity in lung cancer. However, the efficacy of irreversible TKIs in combination with ADCs for the treatment of advanced BC with *HER2*-activating mutations has not been explored yet. Herein, we try to shed light on this question by presenting a case of an estrogen receptor (ER)-positive/HER2-negative ILC with 2 *HER2* activating mutations that showed a significant and durable response to treatment with pyrotinib/T-DM1/exemestane/zoledronic acid.

## Patient Story

**Figure 1.**
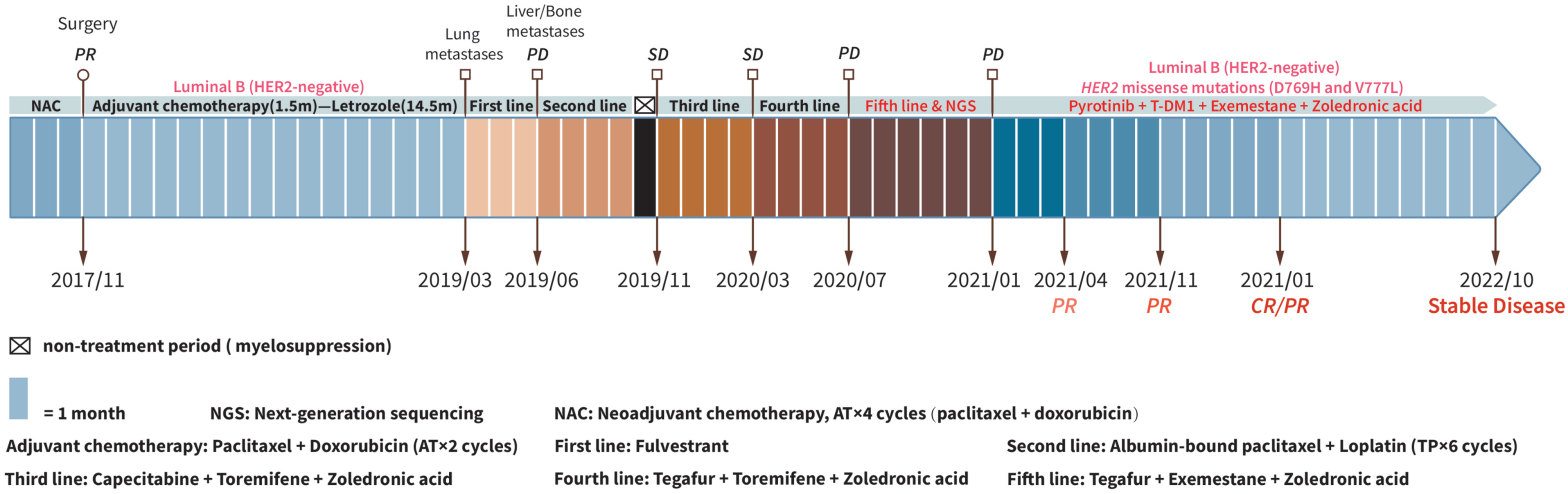
Timeline of therapy and disease response.

A 56-year-old woman was diagnosed with stage II A (cT2N0M0) BC of the left breast in 2017. Core needle biopsy and immunohistochemistry (IHC) staining showed that the tumor was ER-positive (70%+), progesterone receptor (PR)-negative, HER2-negative (1+), and Ki67-positive (20%+). She received 4 cycles of neoadjuvant chemotherapy with doxorubicin and paclitaxel, to which a partial response was observed, and this was followed by modified radical mastectomy (Fig. 1). Postoperative pathological examination confirmed ILC with no axillary lymph node involvement (0/14), Miller-Payne grade 3, and non-pCR. IHC revealed that the resected tumor was ER-positive (70%+), PR-positive (10%+), HER2-negative (1+), and Ki67-positive (5%+). After surgery, she received 2 cycles of adjuvant chemotherapy with doxorubicin and paclitaxel, and this was followed by letrozole for 16 months.

**Figure 2.**
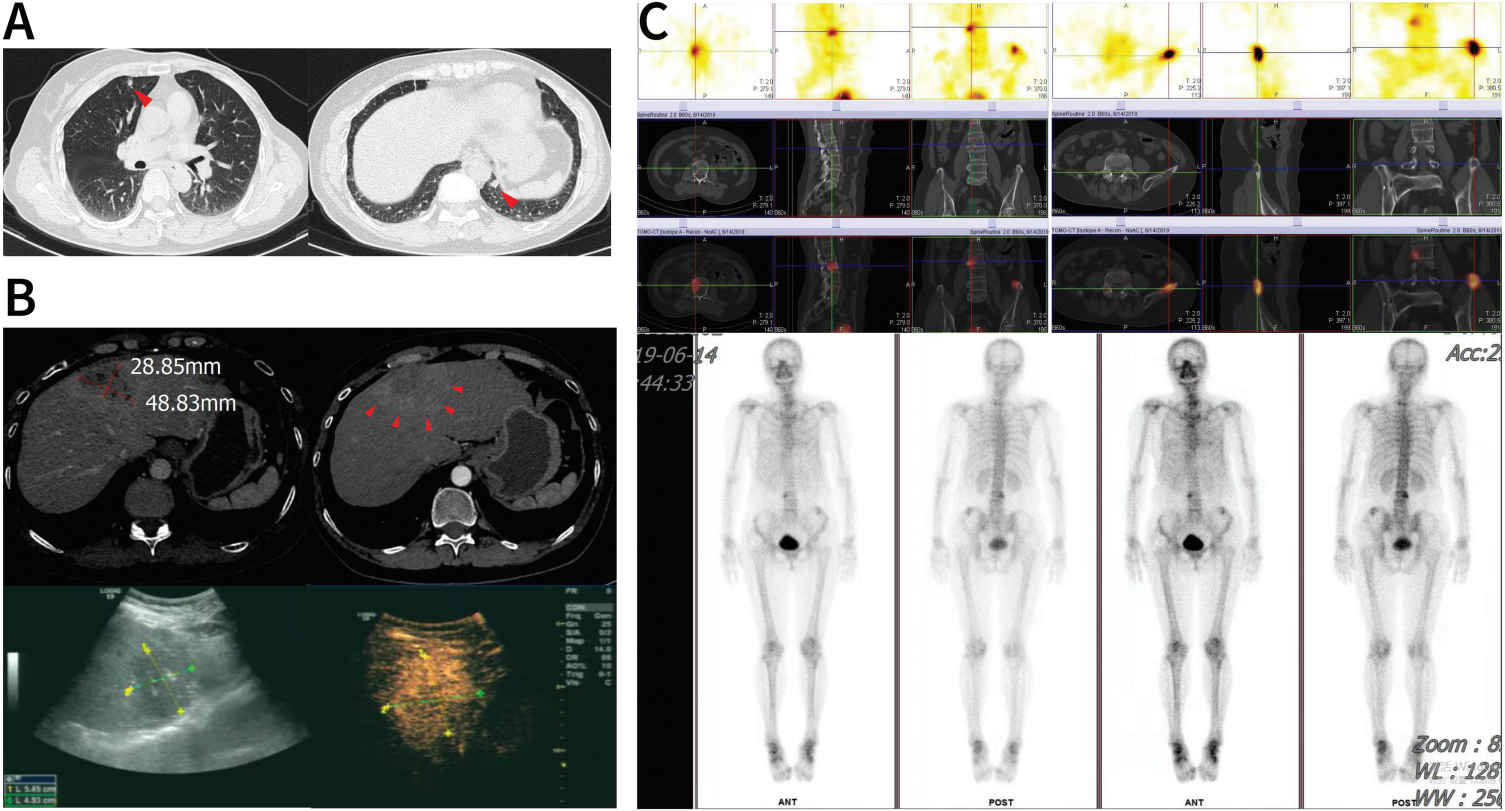
Representative images of lung, liver, and bone metastases at an early stage of systemic metastasis. **A**: Two lung metastases on CT scans (arrows); **B**: left liver metastases on CT scans (upper images, arrows) and contrast-enhanced ultrasound scans (lower images, arrows); **C**: bone metastases on whole-body bone scans (hot spots with increased radionuclide uptake can be observed in the first thoracic vertebra, the third lumbar vertebra, and left ilium).

However, 2 metastases were detected in the lungs on a chest CT scan in March 2019 (Fig. 2). Treatment with a CDK4/6 inhibitor was recommended, but the patient was unable to undergo the treatment because she could not afford it. Therefore, letrozole was replaced by fulvestrant for further maintenance treatment. Three months later, she developed dyspnea and bone pain, and more metastases were detected in the liver and bones (Fig. 2). In addition, the lung metastases detected earlier had increased in size, and right pleural effusion was observed. Liver biopsy and IHC confirmed that the metastases had originated in the BC and were ER-positive (60%+), PR-negative, HER2-negative (1+), and Ki67-positive (30%+). Based on the progression of systemic disease and obvious clinical symptoms, the patient received six cycles of salvage chemotherapy (paclitaxel plus lobaplatin) and 4 doses of zoledronic acid between July and October 2019, but she did not respond to the treatment (stable disease).

After salvage chemotherapy, capecitabine/toremifene/zoledronic acid treatment was administered between November 2019 and March 2020. In March 2020, CT surveillance and bone scan revealed that the metastases in the lungs and liver had decreased in size, but the bone metastases had progressed (with new metastases detected). The patient developed intolerable hand-foot syndrome, so capecitabine was replaced by tegafur. She was started on tegafur/toremifene/zoledronic acid treatment in March 2020, but disease progression was observed in July 2020. Between July 2020 and January 2021, the treatment regimen was adjusted to tegafur/exemestane/zoledronic acid, but she still exhibited systemic disease progression over this 7-month treatment period (with newly detected metastases in the lungs and vertebrae). In January 2021, disease progression was confirmed through imaging examinations, and the patient developed dyspnea, abdominal distention, and exacerbation of bone pain.

## Molecular Tumor Board

**Figure 3.**
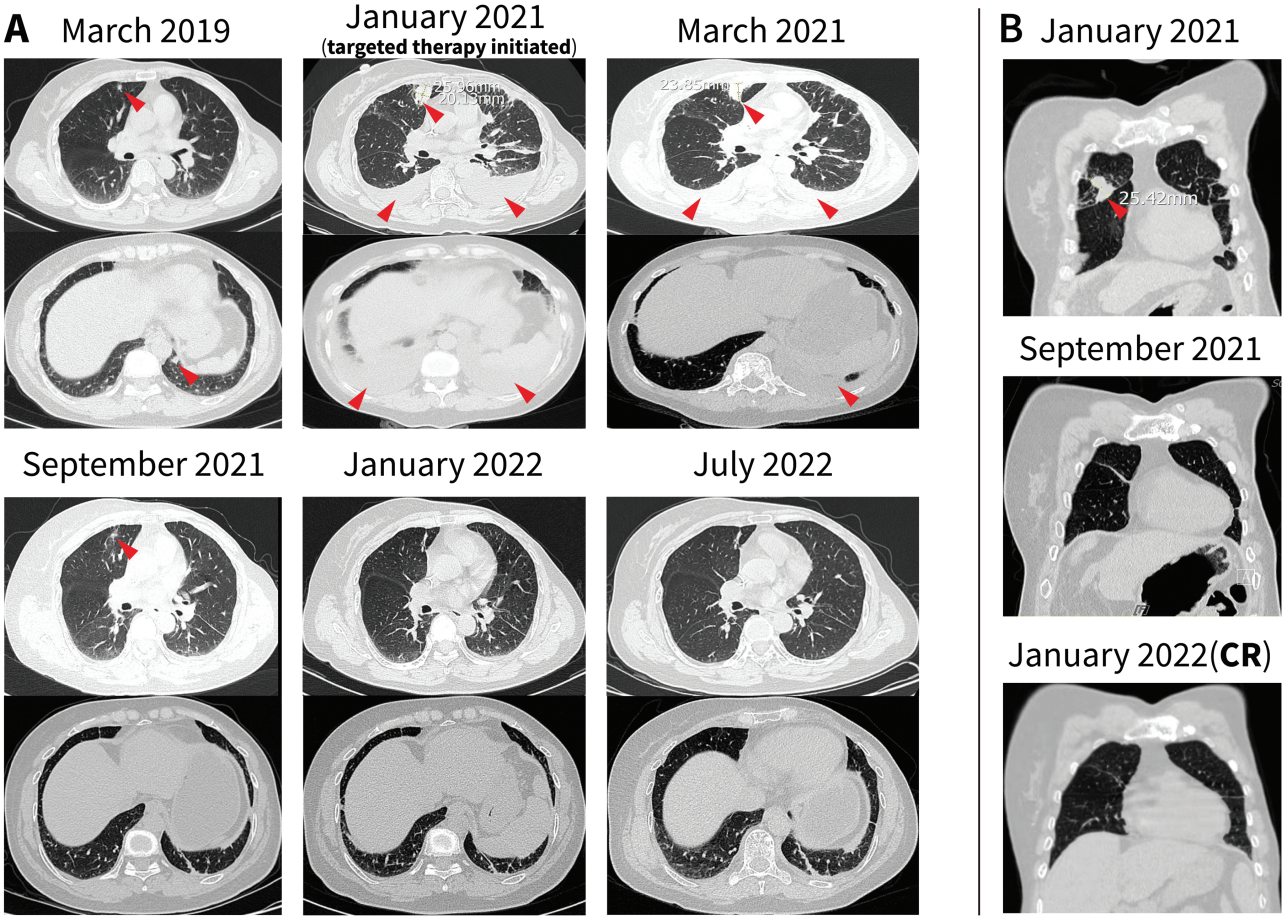
CT scans showing the responses of the lung metastases. **A**: Gradual partial response of the target lesion and pleural effusion in the right and left lung (arrows). **B**: Complete response of the target lesion in the right lung (arrows). CR, complete response; NGS, next-generation sequencing.

Somatic profiling was conducted after discussion with a multidisciplinary team. NGS-based genetic testing (425-gene panel) of the patient’s liver metastatic tissue identified 2 *HER2* missense mutations (D769H and V777L) and a *CDH1* splicing mutation (c.2164 + 1G>C). After careful evaluation and with the informed consent of the patient, pyrotinib/T-DM1/exemestane/zoledronic acid treatment was started in January 2021. Three months after this regimen was started, dyspnea, abdominal distention, and ostalgia almost disappeared, and imaging indicated a decrease in the size of the lung metastases and bilateral pleural effusion (stable disease) (Fig. 3A).

## Patient Update

**Figure 4.**
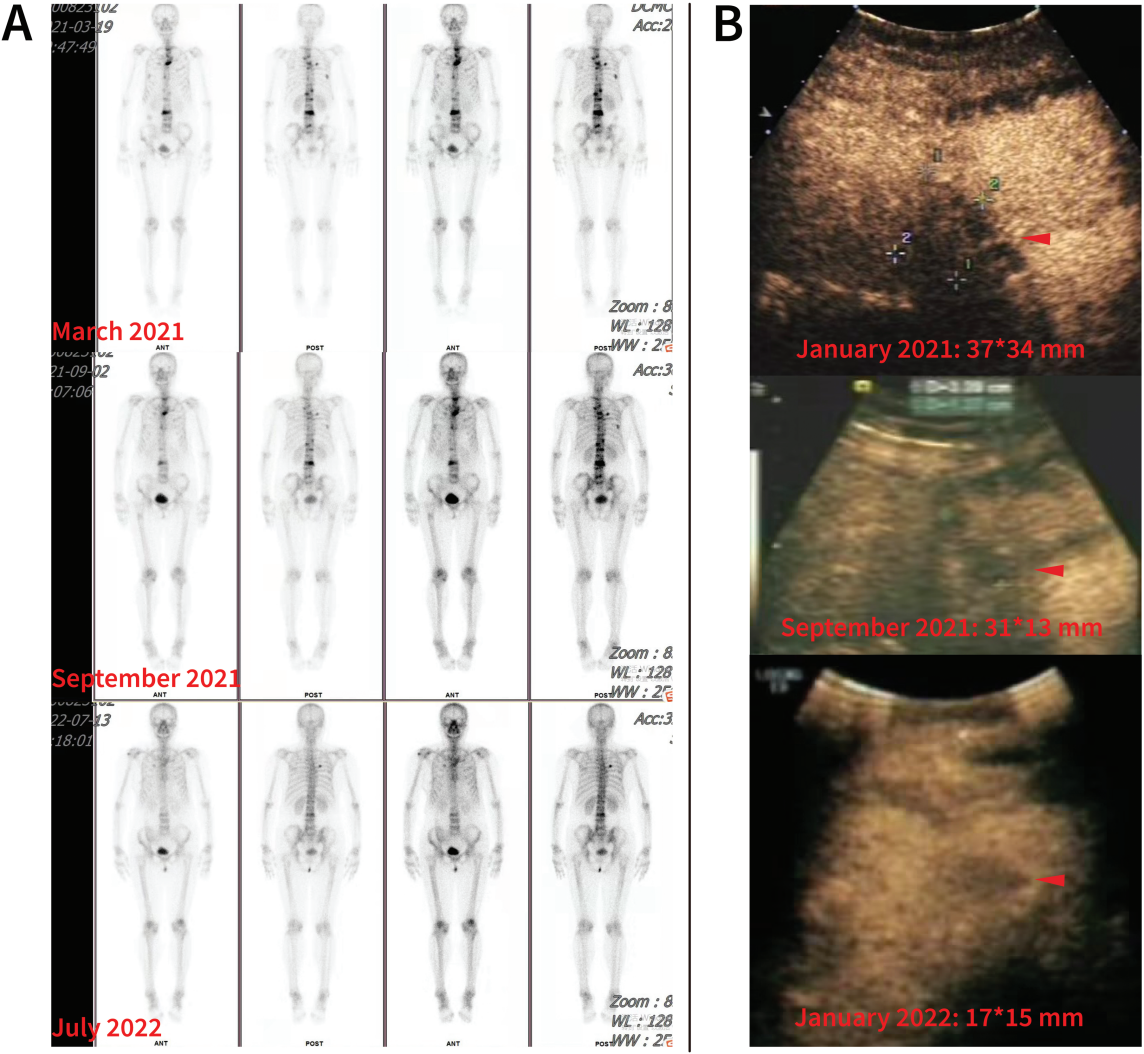
Images showing the responses of bone and liver metastases. **A**: Radionuclide uptake was significantly decreased in bone metastases after the patient received combination therapy for 18 months (July 2022). **B**: The size of the left liver metastases gradually decreased with combination therapy and exhibited a partial response (arrows).

Seven months after the treatment was started, partial response of the lung metastases and almost complete disappearance of the pleural effusion were observed (Fig. 3 A). Twelve months later, the lung metastases were invisible (complete response) (Fig. 3 B), and the liver/bone metastases showed partial remission (Fig. 4). No central nervous system metastases were detected. As of October 2022, the patient had received pyrotinib/T-DM1/exemestane/zoledronic acid therapy for approximately 21 months with tolerable toxicity (grade 2 abdominal distension; grade 1 fatigue, headache, and paresthesia; no myelosuppression occurred), and the disease remained stable after previously achieving partial response.

## Discussion

To the best of the authors’ knowledge, the present report is the first to describe the treatment of ILC carrying *HER2* mutations with combined treatment involving the TKI pyrotinib and the ADC ado-trastuzumab emtansine. The regimen was promising, as the patient achieved stable disease with partial response at 21 months after the treatment was started.

Activating mutations in *HER2*, as well-established oncogenic drivers, have been identified in a vast array of solid tumors.^6,27^ In clinical practice, the efficacy of molecular therapy targeting actionable *HER2* mutations has been mainly investigated in breast and lung cancer.^20,28^ Bose et al functionally characterized 13 *HER2* mutations, and they found that the G309A, D769H, D769Y, V777L, P780 ins, V842I, and R896C mutations are activating mutations that may agonize breast cancer development.^8^ Patients with *HER2*-activating mutations may benefit from anti-HER2 therapy, especially irreversible TKIs. The V777L and D769H mutations are 2 common activating mutations located in the kinase domain, which were identified and considered therapeutic targets for this patient.

The V777L mutation results in an amino acid substitution at position 777 in *HER2*—from a valine (V) to a leucine (L).^29^ Several studies have reported reduced tumor growth and signaling activity in V777L-mutated tumors treated with neratinib and lapatinib.^8,30^ Clinical activities were also observed in *HER2* V777L-mutated cancer treated with neratinib.^20^ Croessmann et al reported that neratinib reverses estrogen-deprivation resistance and fulvestrant resistance in tumors with *HER2* variants and ER-positive tumors with the HER2 missense mutations G309A, L755S, and V777L.^31^ In addition, in a case reported by Lei et al, a patient with HER2-negative advanced BC harboring the V777L mutation was treated with trastuzumab in combination with vinorelbine and had favorable results.^32^ However, a preliminary study indicated that the *HER2* V777L mutation may mediate resistance to trastuzumab in the context of HER2-positive metastatic breast cancer.^33^ The patient in this report harbored not only the V777L mutation but also the D769H mutation, which resulted in an asparagine to histidine substitution at position 769 in the alpha-helix within the kinase domain of *HER*2, affecting ATP binding to HER2 and increasing HER2 dimerization.^8,34^ A preclinical study demonstrated that patients harboring the D769H mutation were sensitive to neratinib as well.^8^ Accordingly, these 2 mutations, together or individually, have been considered as biomarkers in several clinical studies evaluating the efficacy of TKIs or ADCs in BC and other solid tumors (NCT04579380, NCT04209465, NCT04172597, NCT04644237).

Pyrotinib is considered to have potential therapeutic benefits in patients with *HER2* mutations.^35^ A single-arm, prospective, phase-II study on the therapeutic efficacy of pyrotinib in metastatic BC with *HER2* mutations in 10 patients^13^ reported that 1 patient experienced complete response and 3 patients experienced partial response. Further, the median PFS was 4.9 months (95% CI: 3.8-6.0 months), and the clinical benefit rate was 60% (CR+PR+stable disease over 24 weeks). Further, at present, there are many relevant ongoing studies about TKIs for the treatment of *HER2*-mutated BC. For example, there is a phase II, open-label, multicenter study whose principal objectives are to evaluate the efficacy and safety/tolerability of poziotinib in 5 cohorts, including 30 pretreated patients with HER2-positive or HER2-negative BC with *HER2*-activating mutations (NCT04172597). However, although recent research studies and clinical trials have shown that TKI monotherapy may have promising outcomes for the treatment of *HER2*-mutated/HER2-negative advanced BC, TKIs have not yet been approved for the treatment of *HER2*-mutated breast cancer because their clinical activity is limited in terms of the clinical benefit rate, duration of response, PFS, and overall survival.^8,13,19,36^ Moreover, acquired gatekeeper mutations, *PI3K*/*AKT*/*mTOR* pathway activation, and co-occurring mutations in *HER2* and *HER3* confer primary or *de novo* resistance, so these mutations are potential obstacles to the therapeutic efficacy of TKIs.^31,37-39^ Data from the MutHER and SUMMIT trials indicate that patients with ER-positive BC with *HER2* mutations can benefit from dual-blockade therapies, such as neratinib plus fulvestrant, which have demonstrated prolonged PFS and duration of response, as well as a higher clinical benefit rate than neratinib monotherapy (38% versus 30%).^36,40^

T-DM1 is a HER2-targeted ADC in which trastuzumab is linked with the microtubule-inhibitory agent DM1, and it is an approved treatment agent for patients with *HER2*-amplified or overexpressing metastatic BC.^41^ Mukohara et al reported a case of BC with *HER2* amplification and L755S mutation in which the patients exhibited an excellent response to T-DXd, after initial progression with trastuzumab and lapatinib therapy.^42^ Both T-DM1 and T-DXd have been demonstrated to be promising agents for the treatment of lung cancer with *HER2* mutations.^22,42^ At present, the potential of these ADCs for the treatment of *HER2*-mutated metastatic BC is unclear, but there is an ongoing clinical trial on the efficacy of T-DXd for the treatment of *HER2*-mutated BC (NCT04639219).

Combined treatment with TKIs and ADCs in patients with *HER2* mutations is worthy of further exploration. The safety of the T-DM1 plus neratinib regimen has been evaluated in patients with metastatic HER2-positive BC, and 63% (12/19) of patients achieved an objective response with tolerable toxicity.^43^ Further, it has been demonstrated that the irreversible pan-HER TKI poziotinib upregulated HER2 cell-surface expression by decreasing the ubiquitination of mutant HER2 and, consequently, potentiated the antitumor activity of T-DM1.^44^ Notably, in a *HER-*mutated patient-derived tumor xenograft mouse model, combined treatment with poziotinib and T-DM1 resulted in complete tumor regression.^44^ However, Li et al reported that HER2 ubiquitination and internalization, rather than HER2 overexpression, are key mechanisms that mediate endocytosis and the efficacy of ADCs (T-DM1 and T-DXd).^45^ Furthermore, in the context of combination therapy, the irreversible TKIs neratinib and afatinib, instead of reversible TKIs, enhanced receptor ubiquitination, and consequent ADC internalization and efficacy, respectively.^45^ Interestingly, there is a convergence between these 2 studies in terms of phenotypic outcome. Further research is warranted to explain this phenomenon.

In the present case, pyrotinib was chosen as an anti-HER2 agent. This decision was based on the 2 activating mutations (D769H and V777L) in *HER2* identified in this patient, the promising efficacy of TKIs reported in patients with *HER2* activating mutations, the lack of response to previous multiline therapies in this patient, and practical concerns such as drug accessibility (T-DXd was unavailable in China). Although ADCs have not currently been approved for the treatment of *HER2*-mutated metastatic BC, based on the previously reported clinical activity of ADCs alone and ADCs in combination with irreversible TKIs in *HER2*-mutated lung cancer and the findings of ongoing trials evaluating ADC efficacy in metastatic BC with *HER2* mutations, we speculated that our patient would benefit from the addition of T-DM1 to her regimen. Further, exemestane was also added to her regimen because previous studies have shown that simultaneous blockage of ER signaling can improve the prognosis of patients with *HER2*-mutated metastatic BC. This patient responded well to combined therapy with pyrotinib/T-DM1/exemestane/zoledronic acid, with durable PFS. Thus, based on the available evidence and our experience with this patient, we believe that this is currently an ideal combination therapy for patients who have metastatic BC with *HER2*-activating mutations.

The findings of the present case indicate that combined treatment with TKI and ADC is a potent strategy for the treatment of *HER2*-mutated advanced BC. Ongoing trials on this treatment strategy are expected to provide further evidence on the application of this combination regimen and how it can be tailored to patients’ needs based on detected mutations or responses to other lines of treatment. Further, this report also highlights the need to use NGS in challenging cases to detect target mutations, as this can help clinicians make the right treatment decision and lead to better outcomes.

## Data Availability

The authors declare that data supporting the findings of this study are available within the article.
